# Increased red blood cell deformation in children and adolescents after SARS-CoV-2 infection

**DOI:** 10.1038/s41598-023-35692-6

**Published:** 2023-06-17

**Authors:** Julian Eder, Leonie Schumm, Jakob P. Armann, Milo A. Puhan, Felix Beuschlein, Clemens Kirschbaum, Reinhard Berner, Nicole Toepfner

**Affiliations:** 1 Biopsychology, Technische Universität Dresden, Dresden, Germany; 2 Department of Paediatrics, University Hospital and Medical Faculty Carl Gustav Carus, Technische Universität Dresden, Dresden, Germany; 3 Epidemiology, Biostatistics and Prevention Institute, University of Zurich, Zurich, Switzerland; 4 Department of Endocrinology, Diabetology and Clinical Nutrition, University Hospital Zurich, Zurich, Switzerland

**Keywords:** Viral infection, Biomarkers, Paediatric research, Preclinical research, Clinical trial design, Translational research, Biological techniques, Biophysics, Biotechnology, Cell biology, Immunology, Physiology, Biomarkers, Diseases, Medical research, Molecular medicine, Pathogenesis, Physics, Applied physics, Biological physics, Fluid dynamics, Information theory and computation, Optical physics, Techniques and instrumentation

## Abstract

Severe coronavirus disease 2019 (COVID-19) is associated with hyperinflammation, hypercoagulability and hypoxia. Red blood cells (RBCs) play a key role in microcirculation and hypoxemia and are therefore of special interest in COVID-19 pathophysiology. While this novel disease has claimed the lives of many older patients, it often goes unnoticed or with mild symptoms in children. This study aimed to investigate morphological and mechanical characteristics of RBCs after SARS-CoV-2 infection in children and adolescents by real-time deformability-cytometry (RT-DC), to investigate the relationship between alterations of RBCs and clinical course of COVID-19. Full blood of 121 students from secondary schools in Saxony, Germany, was analyzed. SARS-CoV-2-serostatus was acquired at the same time. Median RBC deformation was significantly increased in SARS-CoV-2-seropositive compared to seronegative children and adolescents, but no difference could be detected when the infection dated back more than 6 months. Median RBC area was the same in seropositive and seronegative adolescents. Our findings of increased median RBC deformation in SARS-CoV-2 seropositive children and adolescents until 6 months post COVID-19 could potentially serve as a progression parameter in the clinical course of the disease with an increased RBC deformation pointing towards a mild course of COVID-19.

## Introduction

An infection with the novel severe acute respiratory syndrome coronavirus 2 (SARS-CoV-2) may lead to severe coronavirus disease 2019 (COVID-19) with multi-organ failure and death. Underlying pathomechanisms include a hyperinflammatory response with cytokine storm^1,2^, endothelial damage^3^ and hypercoagulability with the risk of microthrombosis and pulmonary embolism^4^. A common clinical finding is an acute respiratory distress syndrome (ARDS) leading to severe hypoxemia. However, in contrast to sepsis, microcirculation is maintained and even enhanced in COVID-19 patients as a compensatory response^5^. As this mechanism is dependent on red blood cells (RBCs) as one of the main actors in microcirculation and tissue oxygenation, COVID-19-induced changes of RBC properties may have a huge impact on microcirculation. So far, several changes in number and morphology of peripheral blood cells have been described in COVID-19^6–10^. For RBCs specifically, MCV, MCH and RBC distribution width (RDW) have been shown to correlate with COVID-19 severity, thus serving as a prognostic marker for the course of the disease^11–13^. Besides analyzing RBC morphology, there is increasing interest in characterizing RBC mechanical properties related to COVID-19 as these are closely linked to cellular functionality and can thus serve as disease markers^14^. Nader et al. described an increased blood viscosity despite reduced hematocrit and an increased RBC aggregation^15^. Another factor influencing microcirculation is the RBCs ability to pass small capillaries, which in turn depends on their deformability. In association with COVID-19, Piagnerelli et al. report that RBC deformability is not altered in patients with severe ARDS in contrast to patients with bacterial sepsis which may explain the intact microcirculation observed during acute severe COVID-19^5,16^. In contrast, reduced RBC deformability was observed in patients that recovered from mild COVID-19^17^.

A new technique to characterize peripheral blood cells is real-time deformability cytometry (RT-DC), a high-throughput device by exerting shear stress and pressure gradients on cells at a constant flow in a microfluidic channel^18^. RT-DC is not only able to measure morphological changes of peripheral blood cells but to detect altered mechanical properties associated with specific disease pathologies and thus give quick information additional to the conventional differential blood count used in clinical routine^18,19^. For other viral infections, namely diseases by Epstein-Barr virus, rubella virus and parvovirus B19, alterations of blood cell shape, membrane integrity and deformation have already been described by RT-DC analysis^19–21^. For SARS-CoV-2, there is first data available from RT-DC analysis of peripheral blood cells showing RBCs of adult COVID-19 patients to be more heterogeneous in size and deformation^22^. In children and adolescents, infection with SARS-CoV-2 is often pauci- or asymptomatic despite high viral loads, and if symptomatic, they mostly recover quickly^23–26^. However, some present with severe inflammation weeks after SARS-CoV-2 infection – the so called PIMS (pediatric inflammatory multiorgan syndrome) and even if much rarer than in adults, Long-COVID syndrome does exist also in children^27,28^. Identifying specific cell-mechanical changes of RBCs associated with SARS-CoV-2 infection in children is important to further characterize pathophysiological mechanisms of this novel virus disease and may help to understand and monitor the different clinical courses of COVID-19 in children from asymptomatic to critically ill and prolonged recovery including immunological sequelae. In the present study we examined morphological and mechanical alterations of red blood cells between SARS-CoV-2-seronegative and -seropositive children and adolescents with respect to the time of seroconversion.

## Methods

### Study cohort and sample collection

**Figure 1:**
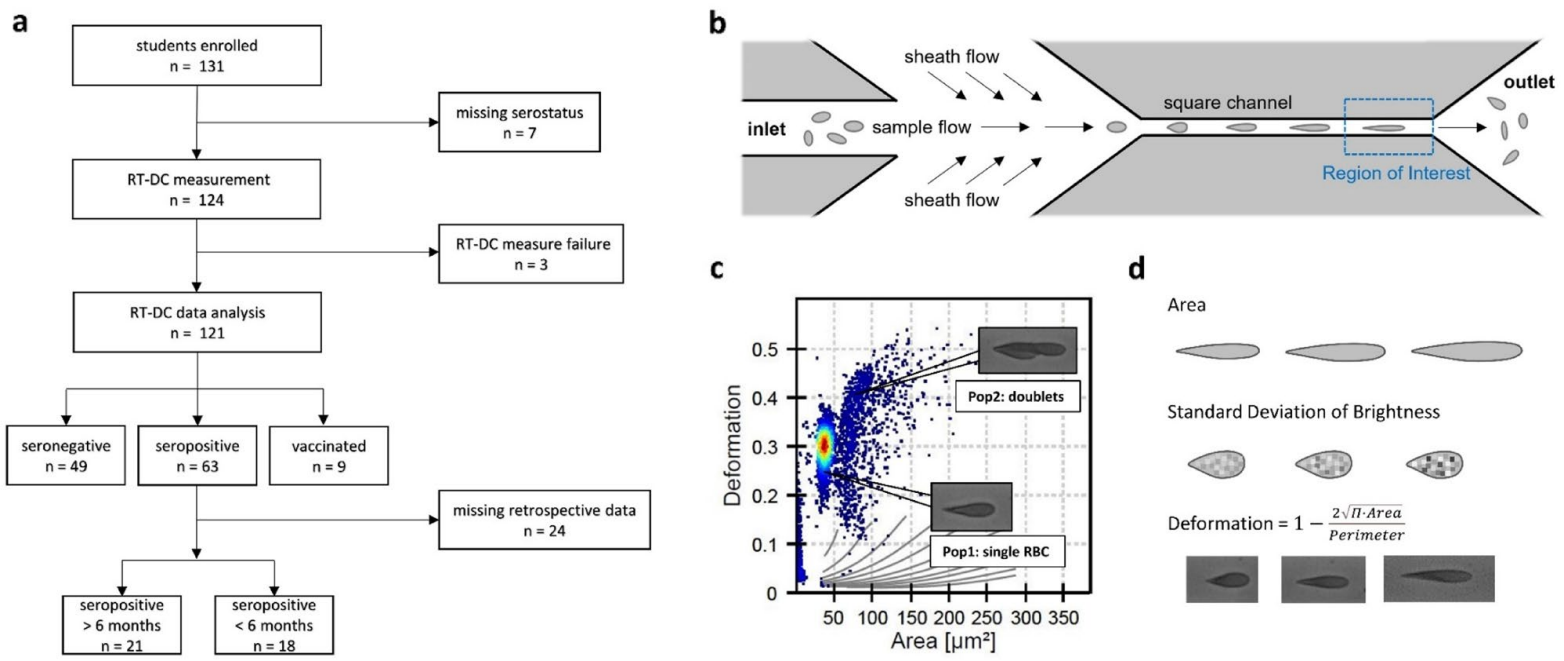
Depiction of the sample flow, real time deformability cytometry measurement and cell classification. Study design and paradigm. (**a**) Upon informed consent 131 students between 11 and 18 years old have been enrolled into the ImmunCOVID19-study. Due to missing information of the serostatus, seven students were excluded. RT-DC of peripheral blood was performed in 124 participants of which three had to be excluded due to measure failure. Results of 121 students were analyzed of which 49 were seronegative and 63 seropositive for SARS-CoV-2-IgG and 9 students had complete status of vaccination. In a subanalysis, seropositive participants were classified according to time of infection. Participants with a positive serostatus (from previous SchoolCoviDD19 study visits) or positive PCR test more than 6 months ago were categorized as “seropositive > 6 months “ (*n* = 21). Participants with negative serostatus in the SchoolCoviDD19 study visits more than 6 months ago or with positive PCR test within the past 6 months were classified “seropositive < 6 months “ (*n* = 18). Due to incomplete retrospective data, 24 subjects had to be excluded from the subgroup analysis (**b**) The illustration shows the operating principle of the RT-DC measurement. Diluted peripheral blood was introduced to the microfluid chip over an inlet. An additional lateral sheath flow created a constant flow of blood cells through the square channel which induced cell deformation through capillary shear stress. Images of the cells were taken with a highspeed microscope at 3000 fps in the last third of the channel (Region of Interest) before the suspension was discharged over an outlet. (**c**) All cells of one RT-DC measurement are plotted according to their deformation and cell size. Population 1 represents intact, single red blood cells (RBCs). Population 2 represents groups of two or more erythrocytes attached to each other. All other populations represent damaged erythrocytes and other cell types such as thrombocytes. Only population 1 was used for further analysis. (**d**) Erythrocytes were analyzed according to area of the cell, standard deviation of brightness and deformation as shown exemplarily for the three parameters. Information on area and standard deviation of brightness were extracted from the cell images in ShapeOut. Deformation was calculated as a ratio of area and perimeter.

Students from fourteen secondary schools in the metropolitan area of Dresden and surrounding rural Saxony, Germany, who already participated in the SchoolCoviDD19-study^29^ with serial assessment of the SARS-CoV-2-serostatus, were invited to participate in the ImmunCoviDD19-study. The study was approved by the ethics committee of the Technical University of Dresden (BO-EK-265052021) and conducted according to the declaration of Helsinki. Upon informed consent of all participants and their legal guardians, 3 ml of peripheral blood were drawn into a sodium citrate S-Monovette® (Sarstedt, Nümbrecht, Germany). Data on demographics and serostatus of all participants was obtained at the same time by the SchoolCoviDD19-study. Upon enrollment, participants had no signs of fever or respiratory tract infections. Between June 24th and July 16th 2021, 131 students were enrolled into the ImmunCoviDD19-study. RT-DC analysis was successfully performed for 121 participants after 10 students had to be excluded because of incomplete data or measure failure. Out of 121 participants, 49 participants were seronegative and 63 seropositive for SARS-CoV-2 IgG. Seropositivity against SARS-CoV-2 was defined as the presence of anti-S1 and anti-S2 specific IgG antibodies > 15.0 AU/ml to SARS-CoV-2 in Diasorin LIAISON® SARS-CoV-2 S1/S2 IgG Assay^29^. In addition, 9 participants reported a complete vaccination status (Fig. 1a).

Retrospective data from previous surveys of the SchoolCoviDD19-study in the same cohort allowed further exploratory subgroup analyses of all seropositive participants with respect to the time of seroconversion. Participants that were tested seropositive in any blood analysis of the SchoolCoviDD19-study before December 10th 2020 or reported a positive PCR before that day were categorized as seropositive with a time of seroconversion more than 6 months ago (seropositive > 6 months). Those who were seronegative until December 10th 2020 or who reported a positive PCR later than December 10th 2020 were categorized as seropositive with a time of seroconversion within the past 6 months (seropositive < 6 months). Twenty-four seropositive participants were not included in the subgroup analysis because of missing information on previous serostatus or PCR testing. Furthermore, subjects, who reported full COVID-19 vaccination at the time of blood collection were categorized as vaccinated. Subjects with a single vaccination dose were not included in this study (Fig. 1a).

### Real-time deformability cytometry (RT-DC) measurements

Measurements of the blood samples were done with the AcCellerator instrument (Zellmechanik Dresden, Dresden, Germany) as described previously^18,19^. For maximum quality, measurements were done no longer than 4 h after blood collection to minimize cell lysis. In preparation for the measurement, citrate blood was diluted 1:20 with phosphate buffered saline (PBS) containing methylcellulose and viscosity was adjusted to 60 mPa s at 24 °C using a falling ball viscometer (Haake, Thermo Scientific). 1 ml of cell suspension was loaded into a syringe, placed in a syringe pump and connected to the microfluid chip made of polydimethylsiloxane (PDMS) attached to cover glass. The inlet of the chip led to a square channel of 20 × 20 µm and 300 µm length. Pure measurement buffer administered via a second inlet to the microfluidic chip induced a focused constant flow of the cell suspension through the channel. The total flow rate was 0.02 µL/s, of which the sheath flow rate was 0.015 µL/s and the sample flow rate was 0.005 µL/s. Images of the cells were taken at the last third of the channel with a high-speed microscope at 3000 fps in a region of 250 × 80 pixels. Cell analysis was focused on RBCs applying filters for object length and heights from 1.0 µm to 80.0 µm and limited to 10 000 events (Fig. 1b).

### Data analysis

Cell images were analyzed using ShapeOut software and custom-written Python scripts (Fig. 1c). For RBCs, median and interquartile range (IQR) of area, deformation and standard deviation of brightness were analyzed as described in-depth previously^30^. In brief, definitions of the parameters were as follows: Area was calculated from all pixels within the cell contour of the convex hull area. Calculation of the deformation parameter reflects the deviation of cell contour from an ideal circle, as depicted in Fig. 1d. In general, brightness takes into account the average brightness intensity of all pixels within a cell, whereby the standard deviation of the average brightness parameter was used to get more information of the within variance and distribution of intensity. Age and gender were used as confounding variables.

### Statistical analysis

Statistical analyses were done in R v4.0.2 (RStudio v 2022.2.3.492)^31^. Two-tailed Welch’s *t*-tests were used to compare median and IQR of erythrocyte deformation, brightness and area between SARS-CoV-2-seropositive and SARS-CoV-2-seronegative participants as well as participants vaccinated against COVID-19. If the data was not normally distributed within a single group of *n* ≥ 30 subjects^32^, statistical tests were conducted independently of variance of the residuals^33^ or normal distribution. The same procedure was applied to mean comparison tests regarding time of SARS-CoV-2-seroconversion (seronegative, seropositive < 6 months, seropositive > 6 months) and cell parameters. To indicate the effect size for Welch’s *t*-tests, Cohen´s d^34^ was used (RStudio: method = “unequal” in cohensD). Moreover, partial correlations between either groups by SARS-CoV-2-serostatus or groups by time of SARS-CoV-2-seroconversion and (median and IQR of) brightness, area and deformation were performed by adding age and gender as possible confounding variables. To control for type 1 errors, *p*-values were adjusted using Holm-Bonferroni correction for multiple comparisons for the number of tests applied to each dependent variable^35^. For all statistical analyses, the level of significance was set to *p* < .05 (two-sided). Data analysis was done in a correlational, exploratory manner.

## Results

In this study, RT-DC analysis was performed for *n* = 121 participants with a mean age of 14.93 (*SD* = 1.82). Thereof, *n* = 63 subjects were seropositive for SARS-CoV-2 and *n* = 49 were seronegative with no differences in age and gender between the two groups. Nine participants reported complete COVID-19 vaccination status at the time of blood collection and were older than SARS-CoV-2 seronegative participants. Descriptive statistics and group comparisons are shown in Table 1.

**Table 1:** Sample characteristics (flow rate = 0.02 µl/s).

	*n*	Min	Max	*M* (*SD*)	*n*	Min	Max	*M* (*SD*)	Test statistic (*df*)	*p*	*p*_*corrected*_
	Seronegative (*n* = 49)				Seropositive (*n* = 63)						
Sex									χ^2^ = 1.2721	.2594	.5188
Male	18	–	–	–	31	–	–	–			
Female	31	–	–	–	32	–	–	–			
Age	–	11	17	14.69 (1.83)	–	11	18	14.92 (1.83)	*t*(103.25) = − 0.6516	.5161	.5188
	Seronegative (*n* = 49)				Vaccinated (*n* = 9)						
Sex									χ ^2^ = 1.5179e − 30	1	1
Male	18	–	–	–	3	–	–	–			
Female	31	–	–	–	6	–	–	–			
Age		11	17	14.69 (1.83)		13	17	16.22 (1.30)	*t*(14.528) = − 3.0179	.0089**	.0178*
	Seropositive < 6 months (*n* = 18)				Seropositive > 6 months (*n* = 21)						
Sex									χ ^2^ = 0.6653	.4147	.8294
Male	11	–	–	–	9	–	–	–			
Female	7	–	–	–	12	–	–	–			
Age		11	18	15.11 (2.17)		12	18	15.19 (1.69)	*t*(31.988) = − 0.1260	*p* = .9006	*p* = .9006

Min = minimum; Max = maximum. Difference tests were performed with Welch’s *t*-test and Chi-square tests; two-tailed; *p*_*corrected*_ = Holm-Bonferroni correction. *p < .05. **p < .01.

### Red blood cell properties after SARS-CoV-2 infection

**Figure 2:**
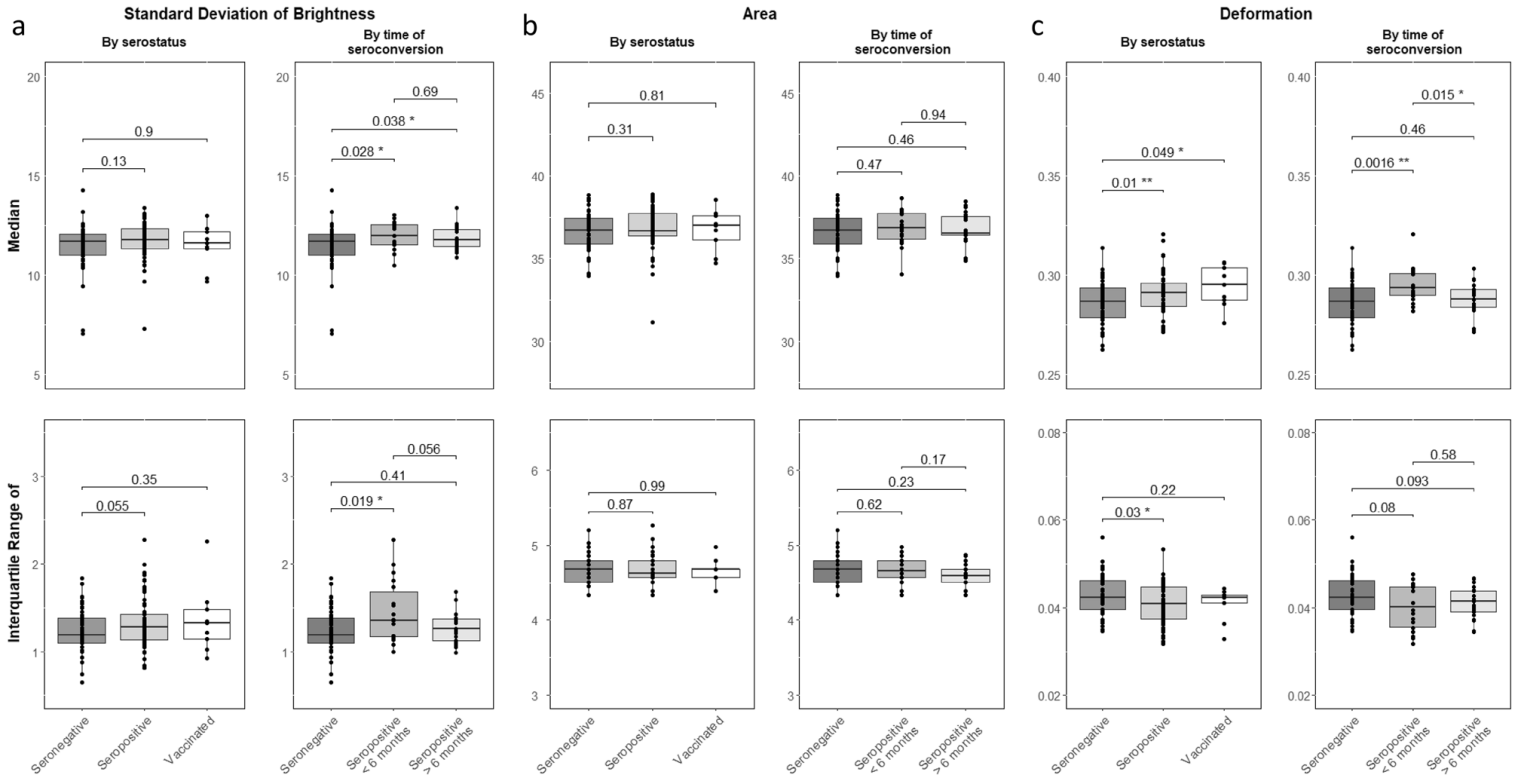
Mean comparison tests of RBC parameters between groups by serostatus and by time of seroconversion. Morphological and mechanical characteristics of RBCs in SARS-CoV-2 seronegative, seropositive and vaccinated participants. (**A**) Brightness of RBCs did not differ between seronegative, seropositive or vaccinated subjects, while in the subanalysis seropositive participants of both subgroups showed brighter RBCs than seronegative participants. SARS-CoV-2 seropositive participants with a seroconversion within the past 6 months had a higher IQR of brightness of the RBCs compared to seronegative subjects. (**B**) RBCs did not differ in size between the analyzed groups. Area was the same for seropositive, seronegative and vaccinated participants, as well as between the subgroups. (**C**) Median deformation of RBCs was higher in seropositive and vaccinated participants compared to seronegative participants. When the time of seroconversion was within the past 6 months, median deformation was significantly higher than in seronegative participants whereas RBC deformation was the same than in seronegative participants when seroconversion was more than 6 months ago. RBC deformation was significantly different between participants with seroconversion within the last 6 months and participants with a seroconversion more than 6 months ago. (Welch’s *t*-test; two-tailed. *p < .05; **p < .01).

For the deformation of RBCs under capillary shear stress, significant differences were found. Median deformation was significantly higher (*t*(102.61) = -2.6109; *p* = .0104, *d* = 0.4978) in SARS-CoV-2 seropositive compared to seronegative individuals while the IQR was lower (*t*(104.31) = 2.1988; *p* = .0301, *d* = 0.4183) (Table 2). Moreover, we found a significant effect in median RBC deformation between SARS-Cov-2-seronegative participants and participants fully vaccinated against COVID-19 (*t*(11.128) = -2.2109; *p* = .0489, *d* = 0.8025) (Supplementary Table 1). No differences in median RBC area, IQR of RBC area, median RBC brightness and IQR of RBC brightness were found between the two groups regarding their SARS-CoV-2-serostatus (Table 2; Fig. 2). With respect to the above-mentioned results, only median RBC deformation survived the application of Holm-Bonferroni correction for multiple comparisons (*p*_*corrected*_ = .0415). Furthermore, we conducted partial correlations between each group comparison and cell parameter and included age and gender as control variables. Partial correlations regarding median RBC deformation remained significant for both main group comparisons after correction for multiple comparisons (seronegative vs. seropositive: *r*_*partial*_ = .2474, *p* = .0992, *p*_*corrected*_ = .0367; seronegative vs. vaccinated: *r*_*partial*_ = .3398, p = .0104, *p*_*corrected*_ = .0367; see Supplementary Table 2). Additionally, a significant negative partial correlation between overall SARS-CoV-2-serostatus and IQR of RBC deformation (*r*_*partial*_ = -.2010, *p* = .0353) as well as a significant positive partial correlation with IQR of brightness (*r*_*partial*_ = .1976, *p* = .0385) was found, which did not survive Holm-Bonferroni correction. No further significant partial correlations were detected regarding median and IQR of RBC brightness and RBC area (see Supplementary Table 2). The complete descriptive data can be found in the corresponding (supplementary) tables.

**Table 2:** Mean comparisons of RBC parameters between seronegative and seropositive participants.

	*M*_*seronevative*_ (*SD*)	*M*_*seropositive*_ (*SD*)	95% CI		*t*	*df*	*p*	*p*_*corrected*_
			LL	UL				
Standard deviation of brightness								
Median	11.4107 (1.1973)	11.7282 (0.9308)	− 0.7330	0.0912	− 1.5474	88.58	.1253	.3759
IQR	1.2290 (0.2430)	1.3265 (0.2885)	− 0.1971	0.0021	− 1.9398	109.26	.0550	.2199
Area								
Median	36.6086 (1.1480)	36.8429 (1.2547)	− .6858	.2173	− 1.0285	107.07	.306	1
IQR	4.6570 (0.1873)	4.6628 (0.1809)	− .07546	.0637	− 0.1631	101.55	.8707	1
Deformation								
Median	0.2858 (0.0106)	0.2911 (0.0104)	− 0.0092	− 0.0013	− 2.6109	102.61	.0104**	.0415*
IQR	0.04275 (0.0045)	0.0408 (0.0046)	0.0002	0.0037	2.1988	104.31	.0301*	.1505

CI = confidence interval; LL = lower limit; UL = upper limit. Difference tests were performed with Welch’s *t*-test; two-tailed;* p*_*corrected*_ = Holm-Bonferroni correction. *p < .05. **p < .01.

### Red blood cell properties in relation to time of SARS-CoV-2-seroconversion

In the subanalysis of seropositive participants regarding the time of SARS-CoV-2-seroconversion, we found that individuals with a seroconversion within the past 6 months exhibit a significantly higher RBC deformation than individuals with a seroconversion more than 6 months ago (*t*(35.099) = 2.5458; *p* = .0155, *d* = 0.8201) (Table 3a). Subsequently, only participants with a SARS-CoV-2-seroconversion within the past 6 months had significantly increased RBC deformation compared to seronegative participants (*t*(34.776) = -3.4231; *p* = .0016, *d* = 0.9118) (Table 3b), while there was no significant difference with respect to median RBC deformation in participants with SARS-CoV-2-seroconversion more than 6 months ago (*t*(46.779) = -0.742; *p* = .4618) (Table 3c). Both effects remained significant after correction for multiple testing (see Tables 3a and 3b). However, no significant difference between the three groups were found regarding IQR of RBC deformation. Interestingly, independent of the time of SARS-CoV-2-seroconversion, both subgroups with a positive SARS-CoV-2-serostatus had significantly brighter RBCs than seronegative subjects (*t*_*seropositive*<*6 months*_(51.249) = -2.2648; *p* = .0278, *d* = 0.5506; *t*_*seropositive*>*6 months*_(66.327) = − 2.1225; *p* = .0375, *d* = 0.4823), while no brightness difference could be detected between the two seropositive subgroups. Regarding the IQR of RBC brightness, a significant difference was found between seronegative and seropositive (seroconversion < 6 months) participants (*t*(23.053) = -2.5203; *p* = .0191, *d* = 0.7512) (Tables 3a–c and Fig. 2). Results did not survive Holm-Bonferroni corrections for multiple testing. Moreover, no significant differences were found regarding median and IQR of RBC area. By calculating partial correlations between groups classified by time of SARS-CoV-2-seroconversion and RBC parameters, significant correlations for median deformation were detected for seronegative compared to seropositive (< 6 months) participants (*r*_*partial*_ = .3776, *p* = .0019) and the two seropositive subgroups (*r*_*partial*_ = -.3690, *p* = .0246). Both reported results survived Holm-Bonferroni correction (seronegative vs. seropositive < 6 months: *p*_*corrected*_ = .0097; seropositive < 6 months vs. > 6 months: *p*_*corrected*_ = .0492) (see Supplementary Table 3). Moreover, a significant positive correlation for IQR of RBC brightness and seronegative compared to seropositive (< than 6 months) participants (*r*_*partial*_ = .3603, *p* = .0032) was found, which survived Holm-Bonferroni correction (*p*_*corrected*_ = .0160). In addition, a significant negative correlation between IQR of RBC brightness and the two seropositive subgroups (*r*_*partial*_ = -.3484, *p* = .0346) was revealed, which did not remain significant after correction for multiple testing. No significant correlations were found between median RBC brightness, median RBC area and IQR of RBC area and the three subgroups after controlling for age and gender.

**Table 3:** Mean comparisons of RBC parameters between subjects regarding their status and time of seroconversion.

	*M*_*seropositve* < *6 months*_* (SD)*	*M*_*seropositve* > *6 months*_* (SD)*	95% CI		*t*	*df*	*p*	*p*_*corrected*_
			LL	UL				
(a) Comparison of seropositive subjects (time of seroconversion: within last 6 months vs. more than 6 months)								
Standard deviation of brightness								
Median	11.9493 (0.7101)	11.8634 (0.5961)	− 0.3452	0.5170	0.4051	33.386	.688	1
IQR	1.4583 (0.3567)	1.2723 (0.1766)	− 0.0049	0.3769	2.0105	23.995	.0558	.2199
Area								
Median	36.8282 (1.0765)	36.8048 (0.9520)	− 0.6428	0.6896	0.0713	34.322	.9435	1
IQR	4.6818 (0.1795)	4.6075 (0.1428)	− 0.0327	0.1813	1.414	32.358	.1669	.8345
Deformation								
Median	0.2948 (0.0091)	0.2876 (0.0085)	0.0015	0.0130	2.5458	35.099	.0155*	.0463*
IQR	0.0402 (0.0053)	0.0410 (0.0035)	− 0.0038	0.0022	− 0.5604	29.053	.5795	.5795

	*M*_*seronegative*_ (*SD*)	*M*_*seropositve* < *6 months*_ (*SD*)	95% CI		*t*	*df*	*p*	*p*_*corrected*_
			LL	UL				
(b) Comparison of seronegative and seropositive (within last 6 months) subjects								
Standard deviation of brightness								
Median	11.4073 (1.1973)	11.9493 (0.7101)	− 1.0224	− 0.0616	− 2.2648	51.249	.0278*	.1390
IQR	1.2290 (0.2430)	1.4583 (0.3567)	− 0.4174	− 0.0411	− 2.5203	23.053	.0191*	.0955
Area								
Median	36.6086 (1.1480)	36.8282 (1.0765)	− 0.8348	0.3956	− 0.7269	32.183	.4726	1
IQR	4.6570 (0.1873)	4.6818 (0.1795)	− 0.1268	0.0773	− 0.4948	31.527	.6242	1
Deformation								
Median	0.2858 (0.0106)	0.2948 (0.0091)	− 0.0143	− 0.0037	− 3.4231	34.776	.0016**	.0080**
IQR	0.0428 (0.0045)	0.0402 (0.0053)	− 0.0003	0.0054	1.8206	26.864	.0798	.3193

	*M*_*seronegative*_ (*SD*)	*M*_*seropositve* > *6 months*_ (*SD*)	95% CI		*t*	*df*	*p*	*p*_*corrected*_
			LL	UL				
Comparison of seronegative and seropositive (more than 6 months) subjects								
Standard deviation of brightness								
Median	11.4073 (1.1973)	11.8634 (0.5961)	− 0.8851	− 0.0271	− 2.1225	66.327	.0375*	.1501
IQR	1.2290 (0.2430)	1.2723 (0.1766)	− 0.1474	0.0608	− 0.8347	51.492	.4077	.6926
Area								
Median	36.6086 (1.1480)	36.8048 (0.9520)	− 0.7292	0.3368	− 0.7413	45.356	.4623	1
IQR	4.6570 (0.1873)	4.6075 (0.1428)	− 0.0330	0.1321	1.206	49.198	.2336	.9344
Deformation								
Median	0.2858 (0.0106)	0.2876 (0.0085)	− 0.0065	0.0030	− 0.742	46.779	.4618	.4618
IQR	0.0428 (0.0045)	0.0410 (0.0035)	− 0.0003	0.0038	1.7133	48.134	.0931	.3193

CI = confidence interval; LL = lower limit; UL = upper limit. Difference tests were performed with Welch’s *t*-test; two-tailed; *p*_corrected_ = Holm-Bonferroni correction. *p < .05. **p < .01.

## Discussion

In this study, we found significant alterations of RBCs after SARS-CoV-2 infection in children and adolescents and could identify differences according to time since SARS-CoV-2-seroconversion. Median RBC deformation was significantly higher in SARS-CoV-2-seropositive participants, after controlling for age and gender. Additionally, IQR of RBC deformation showed a clear trend to be lower but did not survive Holm-Bonferroni correction.

As RBC deformability is depending on the cytoskeletal integrity, increased RBC deformation may indicate fluidization of the cell membrane as a result of structural damage of membrane proteins and lipids observed in COVID-19 patients^36,37,38^. One of the proteins that coordinates the RBCs shape is the membrane bound protein band-3, which was identified to be the target structure of SARS-CoV-2 during the direct infection of RBCs^39^. Fragmentation of band-3 by invasion of the virus into RBCs may thus explain a loss of membrane integrity and increased deformation of RBCs. This gives evidence of a direct effect of SARS-CoV-2 on RBC deformation^40^. Another aspect to RBC membrane modification is an immune complex deposition on the RBC membrane in COVID-19 patients^41^. Binding complement proteins on the surface may alter the RBCs’ membrane properties and thus increase their deformability. Furthermore, mechanical damage of RBCs through capillary shear stress may be increased in case of inflammation of the endothelium in COVID-19 and explains increased deformation^3^. The release of NO from endothelial cells to prevent vasoconstriction and microthrombosis is additionally able to increase RBC deformability and thus their ability to pass small capillaries because of reduced shear resistance^42^. All mechanisms that result in increased RBC deformation may not only be the pathophysiological effect of the infection and inflammation but physiological compensations to prevent microthrombosis and hypoxemia due to SARS-CoV-2 infection. Accordingly, microcirculation was found to be improved during acute COVID-19 as a response to hypoxemia^5^ and RBCs showed a normal deformation compared to patients with bacterial sepsis and reduced deformation^16^. Our finding of increased RBC deformation in seropositive children and young adults with asymptomatic to mild COVID-19 might represent the maximum capacity of a young and healthy organism for these compensatory mechanisms.

This aspect is further supported by our finding that median RBC area is the same in SARS-CoV-2-seropositive and seronegative participants. From previous studies we know that COVID-19 severity correlates with MCV, MCH and RDW as a result of an increased breakdown and turnover of RBCs^11–13^. Release of premature blood cells from the bone marrow leads to anisocytosis because of significantly smaller and stiffer RBCs^43^. Accordingly, Kubankova et al.^22^ observed an increased standard deviation of RBC deformation with a population of small RBCs with low deformation during acute COVID-19 by RT-DC analysis. Conversely, our finding of no differences in median RBC area makes severe cell death with increased turnover and subsequent anisocytosis in our cohort unlikely and points out once again the compensatory capacity of children and adolescents that is sufficient enough to prevent RBC death and severe COVID-19^44,45^. This capacity seems to decrease with increasing age and higher rates of comorbidities and may explain more severe COVID-19 in adults. Diabetes and smoking have been shown to be associated with reduced deformability of RBCs^46,47^ and sepsis and oxidative stress further enhance it^48–50^. A failure to regenerate these RBC alterations and thus persistent reduced RBC deformation may possibly be one underlying mechanism for the onset of Long-COVID^51,52^.

Interestingly, participants with complete vaccination against SARS-CoV-2 also had significantly increased RBC deformation compared to seronegative participants. This finding further supports the hypothesis that altered RBC deformation might be a direct effect to SARS-CoV-2 spike protein presentation and part of the human immune response against SARS-CoV-2^53–55^. However, this observation needs further validation, due to the small group of COVID-19-vaccinated participants.

Another finding of our study is the increased standard deviation of brightness in RBCs after SARS-CoV-2 infection. As this parameter reflects structural changes within the cells, it may be the result of various implications of SARS-CoV-2 on RBCs such as direct infection of RBCs or changes in hemoglobin^11,39,54,56^.

In the subanalysis based on the time of seroconversion we found that increased median RBC deformation is only present until 6 months after SARS-CoV-2 infection and returns back to levels of seronegative participants. This may be seen as a physiological recovery process after the acute infection considering that RBCs have a life span of approximately 120 days^57^. In line with our finding, Kubankova et al. also found RBC deformation back to normal in recovered COVID-19 patients after 4 to 8 months^22^.

Limitations to our study are the heterogeneity of the participants regarding the time of seroconversion and the rather long time since infection. Therefore, a specific statement on the time course of RBC changes after SARS-CoV-2 infection is difficult and interpretation of RBC changes due to SARS-CoV-2 are extrapolations from our observations after recovery from SARS-CoV-2 infection. However, with its broad recruitment strategy better than with highly standardized time points, this study can be seen as a survey investigation of waning or recovery effects after infection. To conclude on RBC alterations in Long COVID-19, further studies e. g. prospective longitudinal case control studies are needed.

Furthermore, it is important to note that the different techniques used by researchers to measure RBC deformation are not comparable one to one. Ektacytometry, used in Piagnerelli et al. measures RBC deformation by determining elongation index at different levels of shear stress up to 50 Pa while RT-DC determines the deformation from the deviation of the cell contour from an ideal circle with shear stress around 100 Pa^58,59^. Therefore, results always need to be presented with reference to the method used.

In conclusion, SARS-CoV-2-infection in children and adolescents is associated with increased RBC deformation during 6 months after infection. This may represent a compensatory capacity during acute COVID-19 to prevent from severe pathological changes and symptoms due to SARS-CoV-2 infection. Understanding the physiological processes involved in mild pediatric COVID-19 courses with successful recovery will enhance our understanding of the pathomechanisms in severe COVID-19 and may additionally help to better understand the onset of Long-COVID-19-syndrom. This study is the first to show, that RBC deformation in children and adolescents measured with RT-DC can potentially be a read-out parameter for these recently discovered RBC changes and could further be elaborated as a point-of-care test, easy-to-use in clinical diagnostics.

## Supplementary Information

Supplementary Information.

## Data Availability

The data that support the findings of this study are available from the corresponding author upon reasonable request.
