# Supplementary material for: Increased red blood cell deformation in children and adolescents after SARS-CoV-2 infection
Source: Sci Rep. 2023 Jun 17;13:9823. doi: 10.1038/s41598-023-35692-6 (PMC10276822; doi:10.1038/s41598-023-35692-6)
Supplement: Supplementary file 1 — Supplementary Information. [file 41598_2023_35692_MOESM1_ESM.docx]

**Increased Red Blood Cell Deformation in Children and Adolescents After SARS-CoV-2 Infection**

Julian Eder, M.Sc.^1+^ Leonie Schumm^2+^, MD; Jakob P. Armann^2^_,_ MD; Milo A. Puhan^3^, MD, PhD; Felix Beuschlein^4^, MD; Clemens Kirschbaum, PhD^1^; Reinhard Berner^2^, MD; Nicole Toepfner^2^*, MD

^+^ These authors contributed equally to this work.

^1^Biopsychology, Technische Universität Dresden, Dresden, Germany

^2^Department of Paediatrics, University Hospital and Medical Faculty Carl Gustav Carus, Technische Universität Dresden, Dresden, Germany

^3^Epidemiology, Biostatistics and Prevention Institute, University of Zurich, Zurich, Switzerland

^4^ Department of Endocrinology, Diabetology and Clinical Nutrition, University Hospital Zurich, Zurich, Switzerland

*Corresponding author:

Nicole Töpfner

nicole.toepfner@uniklinikum-dresden.de

**Supplementary Material**

Supplementary Table 1

|  |  | *M_seronegative_* (*SD*) | *M_vaccinated_* (*SD*) | 95% CI of the difference | | *t* | *df* | *p* | *p_corrected_* |
| --- | --- | --- | --- | --- | --- | --- | --- | --- | --- |
|  |  |  |  | Lower | Upper |  |  |  |  |
| Standard deviation of brightness | | | |  |  |  |  |  |  |
|  | Median | 11.4073 (1.1973) | 11.4592  (1.1023) | -0.9370 | 0.8333 | -0.1280 | 11.751 | .9003 | 1 |
|  | IQR | 1.2290 (0.2430) | 1.3633 (0.3922) | -0.4395 | 0.1707 | -0.9929 | 9.1606 | .3463 | .6926 |
| Area | |  |  |  |  |  |  |  |  |
|  | Median | 36.6086 (1.1480) | 36.7223 (1.2601) | -1.1108 | 0.8836 | -0.2520 | 10.584 | .8059 | 1 |
|  | IQR | 4.6570  (0.1873) | 4.6561 (0.1634) | -0.1313 | 0.1331 | 0.0151 | 12.188 | .9882 | 1 |
| Deformation | |  |  |  |  |  |  |  |  |
|  | Median | 0.2858  (0.0106) | 0.29432 (0.0106) | -0.0169 | -0.00005 | -2.2109 | 11.128 | .0489* | .0977 |
|  | IQR | 0.0428  (0.0045) | 0.0409 (0.0038) | -0.0013 | 0.0049 | 1.2888 | 12.584 | .2207 | .4414 |

*Mean comparisons of RBC parameters between seronegative and fully vaccinated participants*

*Note*. CI = confidence interval; LL = lower limit; UL = upper limit. Difference tests were performed with Welch’s *t*-test; two-tailed; *p_corrected_* = Holm-Bonferroni correction. **p* < .05. ***p* < .01.

Supplementary Table 2

*Partial correlations between cell parameters and main groups adjusted for gender and age*

|  | | Median standard deviation of brightness | IQR of standard deviation of brightness | Median area | IQR of area | Median deformation | IQR of deformation |
| --- | --- | --- | --- | --- | --- | --- | --- |
| Seronegative vs. seropositive | |  |  |  |  |  |  |
|  | *r_partial_* | .1576 | .1976 | .1002 | .0023 | .2474 | -.2010 |
|  | *t*(*df*)  *p* | *t*(112) = 1.6583;  *p* = .1002 | *t*(112) = 2.0950  *p* = .0385* | *t*(112) = 1.0471  *p* = .2974 | *t*(112) = 0.0240  *p* = .9809 | *t*(112) = 2.6536  *p* = .0092** | *t*(112) = -2.1319  *p* = .0353* |
|  | *p_corrected_* | *p* = .4454 | *p* = .1383 | *p* = 1 | *p* = 1 | *p* = .0367* | *p* = .1764 |
| Seronegative vs. vaccinated | |  |  |  |  |  |  |
|  | *r_partial_* | -.0206 | .2132 | .0303 | -.0071 | .3398 | -.2053 |
|  | *t*(*df*)  *p* | *t*(58) = -0.1515  *p* = .8801 | *t*(58) = 1.6037  *p* = .1146 | *t*(58) = 0.2229  *p* = .8245 | *t*(58) = -0.0522  *p* = .9585 | *t*(58) = 2.6550  *p* = .0104* | *t*(58) = -1.5417  *p* = .1290 |
|  | *p_corrected_* | *p* = 1 | *p* = .2292 | *p* = 1 | *p* = 1 | *p* = .0367* | *p* = .3816 |

*Note*. Partial correlations are based on Pearson correlations; two-tailed; *p_corrected_* = Holm-Bonferroni correction. **p* < .05. ***p* < .01.

Supplementary Table 3

*Partial correlations between cell parameters and subgroups adjusted for gender and age*

|  | | Median standard deviation of brightness | IQR of standard deviation of brightness | Median area | IQR of area | Median deformation | IQR of deformation |
| --- | --- | --- | --- | --- | --- | --- | --- |
| Seronegative vs. seropositive (< 6 months) (*n* = 67) | |  |  |  |  |  |  |
|  | *r_partial_* | .2126 | .3603 | .1065 | .0409 | .3776 | -.2086 |
|  | *t*(*df*)  *p* | *t*(67) = 1.7269  *p* = .0891 | *t*(67) = 3.0655  *p* = .0032** | *t*(67) = 0.8498  *p* = .3987 | *t*(67) = 0.3251  *p* = .7462 | *t*(67) = 3.2364  *p* = .0019** | *t*(67) = -1.6930  *p* = .0954 |
|  | *p_corrected_* | *p* = .4454 | *p* = .0160* | *p* = 1 | *p* = 1 | *p* = .0097** | *p* = .3816 |
| Seronegative vs. seropositive (> 6 months) (*n* = 70) | |  |  |  |  |  |  |
|  | *r_partial_* | .1951 | .1182 | 0.0855 | -0.1374 | .1139 | -.1875 |
|  | *t*(*df*)  *p* | *t*(70) = 1.6159  *p* = .1109 | *t*(70) = 0.9670  *p* = .3371 | *t*(70) = 0.6973  *p* = .4881 | *t*(70) = -1.1270  *p* = .2638 | *t*(70) = 0.9313  *p* = .3551 | *t*(70) = -1.5509  *p* = .1257 |
|  | *p_corrected_* | *p* = .4454 | *p* = .3371 | *p* = 1 | *p* = 1 | *p* = .3551 | *p* = .3816 |
| Seropositive (< 6 months) vs. seropositive (> 6 months) (*n* = 39) | |  |  |  |  |  |  |
|  | *r_partial_* | -.0839 | -.3484 | -.0849 | -.2013 | -.3690 | .1534 |
|  | *t*(*df*)  *p* | *t*(39) = -0.4980  *p* = .6216 | *t*(39) = -2.1991  *p* = .0346* | *t*(39) = -0.5042  *p* = .6173 | *t*(39) = -1.2160  *p* = .2321 | *t*(39) = -2.3489  *p* = .0246* | *t*(39) = 0.9186  *p* = .3646 |
|  | *p_corrected_* | *p* = 1 | *p* = .1383 | *p* = 1 | *p* = 1 | *p* = .0492* | *p* = .3816 |

*Note*. Partial correlations are based on Pearson correlations; two-tailed; *p_corrected_* = Holm-Bonferroni correction. **p* < .05. ***p* < .01.
